# The immunoglobulin heavy chain super enhancer controls class switch recombination in developing B cells

**DOI:** 10.1038/s41598-024-57576-z

**Published:** 2024-03-28

**Authors:** Audrey Dauba, Emmanuelle Näser, Dylan Andrieux, Michel Cogné, Yves Denizot, Ahmed Amine Khamlichi

**Affiliations:** 1 Institut de Pharmacologie Et de Biologie Structurale (IPBS), Université de Toulouse, CNRS, Université Toulouse III - Paul Sabatier (UT3), CNRS UMR5089, 205 Route de Narbonne, BP 64182, 31077 Toulouse, France; 2 MOBIDIC, INSERM U1236, Université de Rennes 1, Rennes, France; 3 UMR CNRS 7276, INSERM U1262, Université de Limoges, CBRS, Limoges, France

**Keywords:** Early B cells, *IgH* locus, Super-enhancer, Class switch recombination, Switch transcription, Immunology, Molecular biology

## Abstract

Class switch recombination (CSR) plays an important role in adaptive immune response by enabling mature B cells to replace the initial IgM by another antibody class (IgG, IgE or IgA). CSR is preceded by transcription of the *IgH* constant genes and is controlled by the super-enhancer 3′ regulatory region (3′RR) in an activation-specific manner. The 3’RR is composed of four enhancers (hs3a, hs1-2, hs3b and hs4). In mature B cells, 3’RR activity correlates with transcription of its enhancers. CSR can also occur in primary developing B cells though at low frequency, but in contrast to mature B cells, the transcriptional elements that regulate the process in developing B cells are ill-known. In particular, the role of the 3’RR in the control of constant genes’ transcription and CSR has not been addressed. Here, by using a mouse line devoid of the 3’RR and a culture system that highly enriches in pro-B cells, we show that the 3’RR activity is indeed required for switch transcription and CSR, though its effect varies in an isotype-specific manner and correlates with transcription of hs4 enhancer only.

## Introduction

The immunoglobulin heavy-chain (*IgH)* locus is a paradigm for complex loci undergoing cell type-specific, developmentally-regulated rearrangements and expression. The locus is controlled by a complex and dynamic interplay of distant *cis*-acting elements throughout B cell development^1,2^. Two recombination events take place at the *IgH* locus. In developing B cells, V(D)J assembly generates the variable region genes encoding antigen binding sites^3^. In antigen-activated mature B cells, CSR enables a change of the heavy-chain constant domain of an IgM to that of IgG, IgE or IgA, thereby acquiring new effector functions^4,5^. CSR relies on various signals received by the B-cell (B-cell receptor, cytokines…) and is mediated by particular sequences called switch sequences located upstream of the constant exons^6^. Transcription of switch regions is a pre-requisite for CSR; it originates from switch promoters, called I promoters^6^, and produces long non-coding RNAs that generate secondary structures such as R loops^7^ and G quadruplexes^8^ that provide the substrate^9–11^ for AID (Activation-Induced cytidine Deaminase), the enzyme that initiates CSR^12,13^.

Switch transcription, also called germline transcription, is regulated by various long-range *cis*-acting elements^6^, including enhancers such as the 3’γ1E, located downstream of *C* *γ1* gene^14^, and insulators such as the 5’hs1RI within the *Cα* gene^15–17^, and the 3’ cluster of CTCF-binding elements (3’CBEs) lying downstream of the *IgH* locus^18–20^. The major control element is the super-enhancer 3’ Regulatory Region (3’RR), composed of four enhancers (hs3a, hs1-2, hs3b and hs4) that act in synergy to activate upstream I promoters in an activation-specific manner^6^. Loss of the 3’RR severely impairs CSR by down-regulating switch transcription^21^. The 3’RR enhancer activity correlates with its transcription into enhancer RNAs (eRNAs)^22–25^.

CSR is not restricted to mature B-cells and can occur at a low frequency in primary developing B-cells^26^, but the transcriptional mechanisms involved are yet ill-known. We have previously reported that deletion of the 5’hs1RI insulator leads to premature activation of a subset of I promoters in primary developing B-cells^16^, indicating that active processes involving transcriptional elements operate to regulate switch transcription at early stages of B-cell development^26^. This raises the question as to whether the master 3’RR is involved in these regulatory processes, particularly because the 3’RR mediates a silencing activity prior to the acquisition of an enhancer activity^23^, pointing to the existence of different developmental stage-dependent mechanisms underlying 3’RR activity^6,26^.

Here, we address the role of the 3’RR in CSR in primary pro-B cells using a mouse line devoid of the 3’RR^21^ and an interleukin 7 (IL7)-based culture system that highly enriches in pro-B cells^27^. We report that the 3’RR is required for switch transcription and CSR in pro-B cells, and that its transcriptional activity is restricted to that of hs4 enhancer. We discuss these findings in a comparative perspective with mature B cells.

## Results

### Deletion of the 3’RR reduces switch transcripts levels in pro-B cells

**Figure 1:**
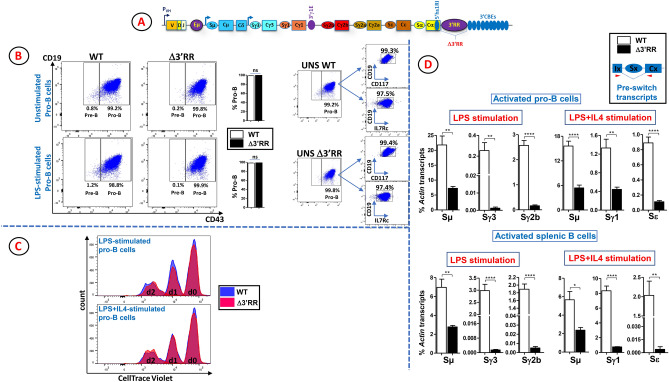
(**A**) Scheme of a rearranged murine *IgH* locus. The black arrow indicates transcription from the promoter of the rearranged V(D)J gene. The known regulatory elements of the locus: Eµ and 3’γ1E enhancers, 5’hs1RI insulator, the 3’RR, and the 10 downstream CTCF-binding elements (CBEs) are shown. The blue arrow indicates the constitutive transcription from Eµ/Iµ enhancer/promoter. The downstream I promoters are signal-dependent. In the Δ3’RR mouse line, the whole 3’RR was deleted. (**B**) B220^+^ cells with the indicated genotypes were sorted and cultured for 5 days in the IL7 medium, then in the presence, or not, of LPS for additional 2 days in the IL7 medium. At day 7, cells were stained with anti-CD19, anti-CD43, anti-CD117, anti-IL7 receptor (IL7Rc), and anti-IgM, and gated on IgM^-^ population. Unstimulated CD19^+^CD43^high^IgM^-^ pro-B cells (left panels) were further checked for CD117 and IL7Rc expression (right panels). (n = 3). (**C**) WT and Δ3’RR B220^+^ cells were sorted and cultured as in (B). At day 5, cells were stimulated with LPS or LPS + IL4 in a CellTrace Violet-containing IL7 medium for additional 2 days. FACS analyses were performed at days 0, 1 and 2. Representative panels are shown for both stimulations (n = 3). (**D**) Quantification of pre-switch transcript levels in in vitro stimulated pro-B cells and splenic B cells with the indicated genotypes. The scheme on the top right represents a constant gene, x stands for any isotype. The relative position of the primers used to detect spliced pre-switch transcripts is indicated. Total RNAs were purified, reverse transcribed and the indicated pre-switch transcript levels were quantified by RT-qPCR (n = 6 for pro-B cells, n = 3 for splenic B cells) (****p < 0.0001, **p < 0.01).

To investigate the potential requirement for the 3’RR in switch transcription in developing B cells, we used a mouse line that harbors a deletion of the whole 3’RR (hereafter Δ3’RR) (Fig. 1A), previously shown to lead to a general defect of CSR in activated mature B cells^21^. Bone marrow B220^+^ cells were propagated in vitro in the presence of IL7 for 5 days. This culture system enables a high enrichment (> 97%) in pro-B cells but kills pre-B cells^27^. Pro-B cells were then stimulated for 2 days with lipopolysaccharide (LPS) alone, which induces Sγ3 and Sγ2b transcription, or with LPS + IL4, which induces Sγ1 and Sε transcription.

We first checked that deletion of the 3’RR did not interfere with pro-B cell enrichment in our culture conditions. We found that the enrichment of unstimulated Δ3’RR pro-B cells was comparable to that of their WT counterparts (> 98%) (Fig. 1B). This pattern did not change when Δ3’RR pro-B cells were stimulated with LPS (Fig. 1B). Because proliferation is required for CSR, we compared the proliferation potential of WT and Δ3’RR pro-B cells upon stimulation with LPS and LPS + IL4. In both stimulation conditions, Δ3’RR pro-B cells proliferated just as well as the WT controls (Fig. 1C). The same held true for AID-deficient pro-B cells (not shown). Therefore, any potential effect of the 3’RR deletion on switch transcription and CSR cannot be ascribed to a defect in cell proliferation.

In both stimulations, deletion of the 3’RR led to ~ threefold reduced levels of Sµ transcripts (Fig. 1D). In LPS-stimulated Δ3’RR pro-B cells, Sγ3 and Sγ2b transcript levels were drastically decreased with a more marked effect on Sγ3 (~ 100-fold) than on Sγ2b (~ 12-fold) (Fig. 1D). Upon LPS + IL4 stimulation, a mild reduction (~ threefold) was seen for Sγ1 transcript levels, while the decrease was more severe for Sε transcript levels (~ 18-fold) (Fig. 1D).

We conclude that deletion of the 3’RR leads to a general decrease of switch transcripts levels, though the effect is milder on Sµ and Sγ1.

### CSR is severely impaired in 3’RR-deleted pro-B cells

To address the role of the 3’RR in CSR, we quantified the levels of post-switch transcripts. These transcripts are produced upon completion of CSR and reflect the efficiency of the process^28^. Because the levels of post-switch transcripts were expected to be low in pro-B cells, we used AID^-/-^ pro-B cells (which are unable to switch) as negative controls providing the background for the qPCR.

**Figure 2:**
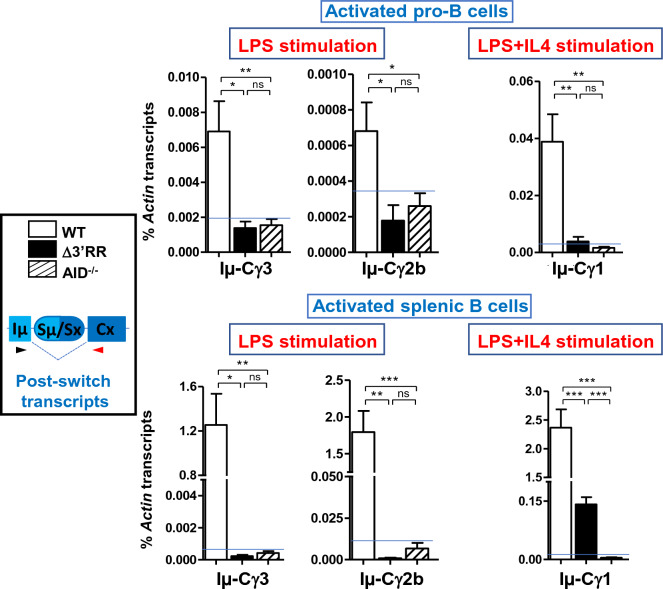
Quantification of post-switch transcript levels in stimulated B220^+^ B cells was as in Fig. 1D. The scheme on the left represents a recombined constant gene. AID-deficient B cells are unable to switch (*i.e.* do not produce post-switch transcripts) and are used as controls for the background level of the qPCR (n = 6 for pro-B cells, n = 3 for splenic B cells) (***p < 0.001; **p < 0.01; *p < 0.05; *ns* not significant).

We found that Iµ-Cγ3 and Iµ-Cγ2b post-switch transcripts were at the background level upon LPS stimulation of mutant pro-B cells, indicating a lack of CSR to Sγ3 and Sγ2b respectively (Fig. 2). Likewise, Iµ-Cγ1 and Iµ-Sε post-switch transcripts were at the background level in Δ3’RR pro-B cells upon LPS + IL4 stimulation (Fig. 2 and data not shown).

Together, these findings strongly suggest that the 3’RR is required for CSR in pro-B cells.

### The 3’RR activity in pro-B cells correlates with hs4 eRNAs production

**Figure 3:**
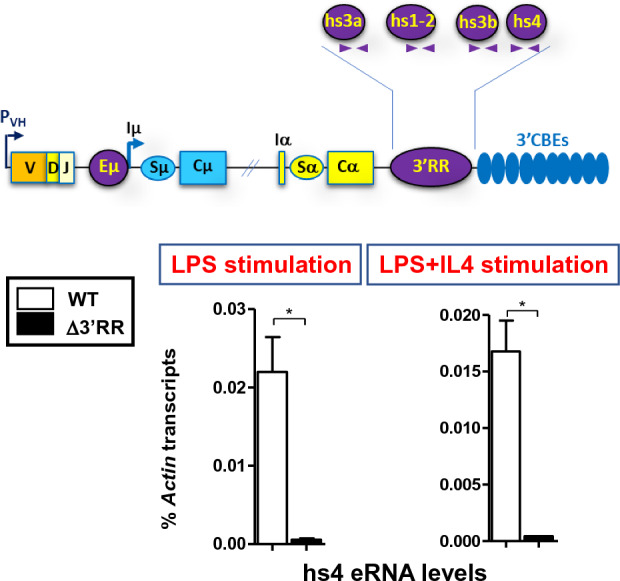
Quantification of eRNAs levels in stimulated pro-B cells was as in Fig. 1D. The top scheme depicts the 3’RR enhancers. The relative position of the primers used to detect 3’RR eRNAs is indicated. Minus RT controls were included throughout (WT, n = 7; Δ3’RR, n = 3) (*p < 0.05).

Enhancer transcription correlates with its activity although there is still a debate on whether transcription per se or the transcript itself (eRNA) that is the crucial element, or whether eRNAs are simply by-products of enhancer activity^6,29^. In activated mature B cells, transcription of the four 3’RR enhancers correlates with 3’RR activity^22–25^. We therefore asked whether the same correlation holds in pro-B cells. To this end, we quantified eRNAs levels in LPS- and LPS + IL4-activated WT pro-B cells. The levels of hs3a, hs1-2 and hs3b eRNAs bordered the background as set up by the Δ3’RR controls (not shown), whereas hs4 eRNAs were readily detectable in both LPS- and LPS + IL4-stimulated WT pro-B cells (Fig. 3). Thus, the 3’RR activity in pro-B cells correlates with hs4 eRNAs production.

## Discussion

In this report, we show that in the context of CSR, the 3’RR, in particular its hs4 enhancer, is active in in vitro activated pro-B cells, and that it regulates switch transcription and CSR. Indeed, in the absence of the 3’RR, we found a general decrease of switch transcript levels though in an isotype-specific manner. Thus, Sµ and Sγ1 transcripts levels were moderately reduced whereas Sγ3, Sγ2b and Sε transcript levels were more severely impacted. In contrast, CSR to all isotypes tested was virtually inhibited as measured by the corresponding post-switch transcript levels.

That the 3’RR is involved in the control of Sµ transcription, driven by the proximal Eµ/Iµ enhancer/promoter, suggests that the latter element alone is not sufficient, and that cooperation between Eµ and 3’RR is necessary for optimal transcription of Sµ region in activated pro-B cells. This observation is reminiscent of the situation in activated mature B cells where Sµ transcript levels were similarly moderately reduced in 3’RR-deleted B cells^21^. Thus, the cooperation between Eµ and the 3’RR appears to be a conserved requirement for the control of Sµ transcription in both pro-B and mature B cells.

With regard to the downstream S regions, loss of the 3’RR in mature B cells drastically impaired switch transcription and CSR to all isotypes except for Sγ1, which was reduced but readily detectable. Likewise, of the downstream isotypes tested in pro-B cells, Sγ1 transcripts levels were the less affected by the 3’RR deletion. Why does Sγ1 transcription relatively escape the stringent control exerted by the 3’RR is still unclear. In mature B cells, this cannot be explained by the activity of the 3’γ1E as its deletion does not affect Sγ1 transcription^14^. It remains to be shown whether 3’γ1E, which displays enhancer activity in pro-B cells^30^, is involved in the control of Sγ1 transcription at this particular developmental stage. Another possibility is that Iγ1 promoter is stronger than the other downstream I promoters.

Regardless, we found that CSR to all isotypes tested was virtually inhibited. This is very likely a consequence of decreased transcription of these isotypes. We note however that inhibition of CSR may also result from the cumulative effect of both reduced Sµ and downstream switch transcription. Overall, our findings suggest that, just as in mature B cells, the 3’RR controls CSR in pro-B cells by regulating switch transcription.

As mentioned previously, the frequency of CSR in developing B cells is lower than in mature B cells^6^. Given the importance of the 3’RR for CSR at both developmental stages, this would suggest that the 3’RR is weaker in pro-B cells than in mature B cells. This suggestion is based on the fact that the four 3’RR enhancers are transcribed in activated mature B cells, potentially resulting in a strong 3’RR enhancer activity, whereas in pro-B cells, only hs4 is transcribed (*i.e.* hs4 eRNAs), at least within the sensitivity limits of our qPCR assay. This would imply that one mechanism through which CSR is checked in developing B cells is the developmental control of the 3’RR strength. But to what extent the 3’RR strength is mechanistically linked to its enhancers’ transcriptional activity (*i.e.* its eRNAs production) remains to be shown.

Seminal studies reported the occurrence of CSR in Abelson murine leukemia virus-transformed pre-B cell lines^*e.g.*^^31–35^, and subsequently in early primary B cells^*e.g.*^^36–40^, including *Rag*-deficient pro-B cells^*e.g.*^^27,41,42^. In the latter context, relatively high levels of γ2b and ε switch transcripts were detected in activated *Rag*-deficient pro-B cells^27,42^. This bias was attributed to a unique three-dimensional chromatin conformation of the *IgH* locus that supports isotype-specific CSR in pro-B cells^42^, and which may involve chromatin loops that form between the 3’RR and the 3’γ1E, and between the 3’γ1E and *γ3* gene^43^. Accordingly, γ3, and to a lesser extent γ1, switch transcript levels were very low to undetectable in activated *Rag*-deficient pro-B cells^27,42^. Interestingly, in activated WT pro-B cells (this study), the levels of γ3 switch transcripts, and to varying degrees γ1, γ2b and ε, were substantially increased compared to their *Rag*-deficient counterparts^27^. Whether this correlates with the disruption of the 3’γ1E-*γ3* loop for instance and/or generation of novel loops in WT pro-B cells is presently unclear. In this regard, a major difference obviously concerns the unrearranged status of the *IgH* variable region in *Rag*-deficient pro-B cells. In contrast, in WT pro-B cells, the large deletions associated with D-J_H_ and V_H_-DJ_H_ recombination events, while they certainly affect the *IgH* locus structure, may impact the long-range interactions that promote switch transcription and CSR. It will be interesting to track these interactions in WT pro-B cells.

In this context, previous studies detected, with some differences, multiple interactions between the 3’RR and various sequences along the *IgH* locus^42–47^ (reviewed in reference ^48^). Surprisingly, individual deletion of the 3’RR or of the core Eµ enhancer, had no apparent effect on long-range interactions across the *IgH* locus in *Rag2*-deficient pro-B cells^45^. This raises interesting questions on the relationship between the dynamics of chromatin architecture and switch transcription and CSR. Thus, a recent model of the long-range mechanisms that control CSR in mature B cells involved cohesin-mediated chromatin loop extrusion, which promotes synapsis of *IgH* enhancers, activated I promoters, S regions, and DNA double-strand break ends necessary for productive CSR in CSR centres (CSRCs)^49^. In the process, Eµ enhancer and the 3’RR act as dynamic impediments to loop extrusion^49^. Thus, one might expect a major effect on CSR in the absence of Eµ or 3’RR. However, while deletion of the 3’RR indeed drastically impaired switch transcription and CSR^21^, deletion of Eµ had at best a moderate effect in Eµ-deleted mice^50^, pointing to additional mechanisms. Somewhat similarly, the 3’CBEs were proposed to play a major role in CSR to Sγ1 as induced Sµ-Sγ1 synapsis was found to be mostly associated with the 3’CBEs rather that the 3’RR^51^. However, deletion of the 3’CBEs in mice had no effect on CSR to IgG1^20^. Clearly, additional work is needed to establish the causal relationship between large-scale chromatin dynamics and the fine details of the induced transcriptional and epigenetic mechanisms that operate during switch transcription and CSR in early and mature B cells. The use of mutant mice devoid of the critical *IgH* regulatory elements such as the 3’RR should be highly informative.

## Materials and methods

### Mice and ethical guidelines

WT 129Sv1 mice were purchased from Charles River. The Δ3’RR mouse line was described in detail in reference 21. AID-deficient mice were provided by T. Honjo, through C-A. Reynaud and J-C. Weill. All the mice were of 129Sv genetic background, and were 6–8 week-old. The experiments on mice were carried according to the CNRS Ethical guidelines and were approved by the Regional Ethical Committee (Accreditation N° F31555005), and complying with ARRIVE guidelines.

### In vitro stimulation of primary medullar and splenic B cells

Single cell suspensions from the bone marrows and spleens were obtained by standard techniques. B220^+^ cell populations from erythrocyte-depleted bone marrows were sorted by using B220-magnetic microbeads and MS columns (Miltenyi), and cultured in the IL7 medium^27^ made up of OPTIMEM supplemented with fetal bovine serum (10%), IL7 (2 ng/ml), β-mercaptoethanol (50 µM), Glutamax (1x) and penicillin/streptomycin (200 U/ml) (all culture medium components were from Fisher Scientific except for IL7 which was from Pepro Tech). For in vitro stimulations, B220^+^ bone marrow cells were grown in the presence of IL7 for 5 days at a density of 3 × 10^5^ cells/ml, then in the presence of LPS (50 µg/ml) or LPS + IL4 (50 µg/ml and 25 ng/ml respectively) and IL7 (2 ng/ml) for additional 2 days. Purification of splenic B cells and stimulation conditions were exactly as described previously^16^.

### Flow cytometry

Sorted B220^+^ BM cells were propagated in the IL7 medium and stimulated or not with LPS as described above. The cells were stained with anti-CD19-APC, anti-CD43-PECy7, CD117-BV711, IL7 receptor-PE, and anti-IgM-FITC, and gated on IgM^-^ population. Pro-B cells were then defined as CD19^+^CD43^high^ and pre-B cells as CD19^+^CD43^low^.

### Cell proliferation assay

The assay was conducted according to the manufacturer’s instructions (Invitrogen). Briefly, B220^+^ bone marrow cells were grown in the presence of IL7 for 5 days as described above. After centrifugation, cell concentration was adjusted to 1 × 10^6^ cells/ml (in a final volume of 6 ml), and the cells were incubated with freshly diluted CellTrace Violet (final concentration 1 µM) at room temperature for 20 min, protected from light. After addition of 30 ml of complete culture medium and incubation at room temperature for 5 min, the cells were pelleted, resuspended in a pre-warmed complete culture medium at a concentration of 1 × 10^6^ cells/ml, and incubated for 10 min at room temperature. The cells were then analyzed by FACS (day 0), or stimulated with LPS or LPS + IL4 as described above, and assayed for proliferation at day 1 and day 2 post-stimulation.

### Quantification of transcript levels by RT-qPCR

Total RNAs were prepared using a commercial kit (Zymo Research), reverse transcribed (Invitrogen), and subjected to qPCR using Sso Fast Eva Green (BioRad). *Actin* transcripts were used for normalization. For eRNAs quantification, minus RT controls were tested for all samples. The primers used to quantify spliced switch transcripts and eRNAs are listed in Table 1.

**Table 1:** Primers used in this study.

Primer	Sequence	Tm °C
Switch transcripts		
Iµ-F	CTCTGGCCCTGCTTATTGTTG	60
Cµ-R	GAAGACATTTGGGAAGGACT	60
Iγ3-F	CAGCCTCAAGGAGATGATGGG	61
Cγ3-R	CAAGGGATAGACAGATGGGGC	61
Iγ1-F	GCACACCCCACAGACAAACC	61
Cγ1-R	ATGGAGTTAGTTTGGGCAGCAG	61
Iγ2b-F	AAGAGTCCAGAGTTCTCACACACAG	60
Cγ2b-R	CCAGTTGTATCTCCACACCCAG	60
Iε-F	TAGAGATTCACAACGCCTGGG	60
Cε-R	CAGGGCTTCAAGGGGTAGAG	59
eRNAs		
hs3a-F	GGCTCCTGTACTGATCGATGG	60
hs3a-R	ACTGTCCCAGTTGCAGCCC	60
hs 1.2-F	GGGTGGCTCAACACCCCAGG	68
hs 1.2-R	GGCTGAGGCAGGCCAAGA	62
hs 3b-F	GAGGGCCAGGGCCCAATGAC	61
hs 3b-R	GGATCTCGGTCCTGGTAACTGGC	66
hs 4-F	CAGGCAAGGTGATGTGGATGAGAG	65
hs 4-R	AGGTCTACACAGGGGCTCTG	56
Normalization		
Actin-F	TACCTCATGAAGATCCTGA	60
Actin-R	TTCATGGATGCCACAGGAT	60

### Statistical analysis

Results are expressed as mean ± SD (GraphPad Prism) and overall differences between values at day 5 from the start of culture and day 7 (*i.e.* day 2 post-stimulation) were evaluated by *t*-test with Mann–Whitney Post-test. The difference between means is significant if *p* value < 0.05 (*), very significant if *p* value < 0.01 (**), extremely significant if *p* value < 0.001 (***) or if *p* value < 0.0001 (****).

## Data Availability

All data generated or analysed during this study are included in this published article.

## Abbreviations

3’RR: 3’ Regulatory region
CSR: Class switch recombination
*IgH*: Immunoglobulin heavy chain
LPS: Lipopolysaccharide
IL: Interleukin
